# Enzymes Enhance Biofilm Removal Efficiency of Cleaners

**DOI:** 10.1128/AAC.00400-16

**Published:** 2016-05-23

**Authors:** Philipp Stiefel, Stefan Mauerhofer, Jana Schneider, Katharina Maniura-Weber, Urs Rosenberg, Qun Ren

**Affiliations:** a Laboratory for Biointerfaces, Empa, Swiss Federal Laboratories for Materials Science and Technology, St. Gallen, Switzerland; b Borer Chemie AG, Zuchwil, Switzerland

## Abstract

Efficient removal of biofilms from medical devices is a big challenge in health care to avoid hospital-acquired infections, especially from delicate devices like flexible endoscopes, which cannot be reprocessed using harsh chemicals or high temperatures. Therefore, milder solutions such as enzymatic cleaners have to be used, which need to be carefully developed to ensure efficacious performance. *In vitro* biofilm in a 96-well-plate system was used to select and optimize the formulation of novel enzymatic cleaners. Removal of the biofilm was quantified by crystal violet staining, while the disinfecting properties were evaluated by a BacTiter-Glo assay. The biofilm removal efficacy of the selected cleaner was further tested by using European standard (EN) for endoscope cleaning EN ISO 15883, and removal of artificial blood soil was investigated by treating TOSI (Test Object Surgical Instrument) cleaning indicators. Using the process described here, a novel enzymatic endoscope cleaner was developed, which removed 95% of Staphylococcus aureus and 90% of Pseudomonas aeruginosa biofilms in the 96-well plate system. With a >99% reduction of CFU and a >90% reduction of extracellular polymeric substances, this cleaner enabled subsequent complete disinfection and fulfilled acceptance criteria of EN ISO 15883. Furthermore, it efficiently removed blood soil and significantly outperformed comparable commercial products. The cleaning performance was stable even after storage of the cleaner for 6 months. It was demonstrated that incorporation of appropriate enzymes into the cleaner enhanced performance significantly.

## INTRODUCTION

Endoscopes are widely used as a valuable diagnostic and therapeutic tool; however, it has been reported that health care-associated outbreaks of infections can be more frequently linked to contaminated endoscopes than to any other medical device (1, 2). Endoscopes are in contact with different body fluids, and the channels provide an ideal surface for bacterial adhesion. Viable bacterial cells can be detected on many endoscopes even after cleaning and disinfection processes (3–6). The main reason for this is that under natural conditions, most bacteria occur in the form of biofilms. They adhere to surfaces and are embedded in a self-produced layer of extracellular polymeric substances (EPS) (7, 8). EPS provide structural integrity to biofilms and protect the bacteria against environmental influences such as UV irradiation, antibiotics, and disinfection and make them much more tolerant to these stresses than planktonic cells (9–11). It is a huge challenge to avoid and remove biofilms, especially in moist environments such as used endoscope channels.

The long and narrow endoscope channels are difficult to reach by mechanical devices, and the use of harsh chemicals or high temperatures could harm the sensitive materials built into endoscopes. For reprocessing of endoscopes, mild cleaning agents are needed to combat biofilms. One effective approach is to destabilize the biofilm EPS, which contain proteins, polysaccharides, lipids, extracellular DNA, and other substances. Some enzymes such as protease (12, 13), DNase I (12, 14), alginate lyase (15, 16), amylase (13, 17), and cellulase (18, 19) have been reported to support biofilm removal. Therefore, inclusion of these enzymes in cleaning agents can improve the efficiency of biofilm detachment. A few enzymatic cleaners are commercially available, but they often failed to show the expected biofilm removal efficacy in practice (20). One of the reasons for failure is the use of inappropriate test parameters during the cleaner development process, which might lead to an overestimation the cleaning performance, e.g., relevance of the used microorganisms, biofilm formation conditions, or readout of biofilm removal.

Here we describe a process for the development and evaluation of novel enzymatic cleaners targeting endoscope biofilms. We selected a biofilm quantification method to assess the cleaners based on methods described in a previous study (21). The performance of newly formulated enzymatic cleaners in the removal of biofilms formed by clinical isolates of Pseudomonas aeruginosa and Staphylococcus aureus was first screened and optimized in a 96-well-plate system. Afterwards, standard methods were used to evaluate the efficacy of biofilm removal from endoscope surfaces and cleaning of coagulated blood. A new cleaner (deconex Prozyme Active) containing four enzymes in a novel base formulation was developed and appeared to perform better than nine comparable commercial products.

## MATERIALS AND METHODS

### Terms and abbreviations.

Base formulations (abbreviations starting with B) are cleaner solutions, including surfactants and other ingredients, without enzymes (B1A and B2B, etc.). Abbreviations starting with E refer to different enzymes, including proteases, polysaccharidases, lipases, and DNases (E1 and E2, etc.). Cleaners (abbreviations starting with C) are commercially available endoscope-cleaning solutions (C1 and C2, etc.). High-level disinfectant is a solution that should achieve complete elimination of all microorganisms in or on an instrument.

### Chemicals and reagents.

Chemicals and reagents were purchased from Sigma-Aldrich (Switzerland) if not mentioned otherwise. Enzyme solutions were obtained from Novozymes (Denmark), and cleaner base formulations were provided by Borer Chemie AG (Switzerland).

### Bacterial strains and growth conditions.

Bacterial strains were obtained from the Leibniz Institute German Collection of Microorganisms and Cell Cultures GmbH (DSMZ). Pseudomonas aeruginosa (DSM 1117) and Staphylococcus aureus (DSM 20231) were grown on tryptic soy agar at 37°C. Liquid cultures were grown in 30% tryptic soy broth (TSB) (9 g/liter, which corresponds to 30% of recommended concentration) supplemented with 2.5 g/liter glucose at 37°C and 160 rpm.

### Biofilm removal assay using 96-well plates.

Bacterial cultures grown overnight were diluted to an optical density at 600 nm (OD_600_) of 0.2 in 30% TSB supplemented with 2.5 g/liter glucose. Two hundred microliters of the bacterial suspension per well was added to transparent (for absorbance) or white (for luminescence) flat-bottom polystyrene 96-well plates (BRANDplates pureGrade). The biofilm in the wells was washed once with 350 μl of a 0.9% NaCl solution before cleaner treatment. All cleaners were used at a concentration of 1% in freshly prepared water of standardized hardness (WSH) containing 1.25 mM MgCl_2_, 2.5 mM CaCl_2_, and 3.33 mM NaHCO_3_ in deionized water. Each column (6 wells with bacteria and 2 wells with medium only) was treated with a different cleaner. A mixture of 1% SDS, 1% EDTA, 1% NaOH, and 0.1% NaClO was used as a positive control, and WSH was used as a negative control. Treatment was done with 250 μl cleaner per well for 40 min at 25°C. To determine the staining background, two rows of the microplate were filled with medium without bacteria. Plates were incubated for 24 h at 33°C with shaking at 40 rpm. For biofilm quantification, crystal violet staining and a BacTiter-Glo assay were applied as described previously (21).

### Cleaning performance against artificial blood soil.

TOSI (Test Object Surgical Instrument) slides (Pereg, Germany) were used by immersing them without a plastic cover in the cleaning solutions to be tested. No mechanical force was applied. The slides were removed from the cleaning solution after 15, 30, and 45 min of incubation; photographed; and then immersed again. At the 60-min time point, the slides were removed, photographed, gently rinsed with deionized water, and photographed again. The resultant cleaning kinetics was judged visually.

### Cleaning performance using EN ISO 15883.

Biofilm was formed in Teflon tubes (Karl Storz, Germany) according to Annex F of part 5 in EN ISO 15883 (version 2005) (https://www.iso.org/obp/ui/#iso:std:iso:ts:15883:-5:ed-1:v1:en). Treatment with a cleaner or WSH (negative control) was done at a flow rate of ∼200 ml/min for 15 min at 25°C. If stated, disinfection after cleaning was done with deconex HLD PA/PA20. The tubes were cut into small pieces, and detachment of biofilms was done by vortexing in a NaCl solution. The following quantifications were conducted: (i) the OD_600_ of the suspension was measured, (ii) viable cells were quantified by determination of CFU on agar plates, (iii) protein levels were quantified using the Lowry assay (22), and (iv) polysaccharide levels were quantified by the phenol-sulfuric acid method (23).

### Statistical analysis.

For each sample, the biofilm value was calculated by subtracting the mean value for the 2 wells with medium only from the arithmetic mean for 6 wells with biofilm. Sample standard deviations were calculated from the values for the 6 similarly treated wells. Statistical significance was determined by using the unpaired, parametric, two-tailed Student *t* test. The value of the negative-control (WSH) wells was set to 100%, and the other values were calculated accordingly. Three independent experiments with six repeats per condition were performed for comparison to commercial products in the 96-well-plate biofilm removal assay.

### Further details.

More detailed materials and methods are provided in the supplemental material.

## RESULTS AND DISCUSSION

### Screening for enzyme-supportive base formulations.

It was found that a small amount of protease alone was sufficient to completely remove S. aureus biofilms, almost independent of the base formulation (data not shown). Therefore, the use of S. aureus as a model microorganism is not appropriate for the selection of a base formulation since the bacteria found on endoscopes include many other species (3), of which, for example, the predominant species P. aeruginosa could not be easily removed with only protease (21).

**FIG 1:**
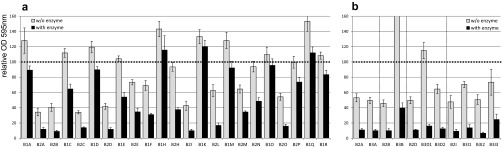
Screening for novel base formulations. Shown is the amount of P. aeruginosa biofilm remaining after treatment with enzyme-free base formulations (light gray) compared to that remaining after treatment with the same base formulations containing a mixture of seven enzymes (dark gray). The *y* axis represents the biofilm amount quantified by crystal violet staining relative to the negative control (biofilm treated with WSH containing no detergents or enzymes). Error bars represent data from 6 individual replicates. In a first round of screening, 4 base formulations were selected (a) and further optimized in a second round of screening (b). B3A is a derivative of B2A, B3B is a derivative of B2B, B3D1/2 is a derivative of B2D, and B3I1/2/3 is a derivative of B2I.

A first round of screening with 23 novel base formulations (surfactants and other ingredients without enzymes) was performed based on the prerequisite that the desired compositions should support enzyme activity and display maximal biofilm removal. For this purpose, P. aeruginosa biofilm was treated with base formulations mixed with or without an enzyme cocktail (Fig. 1a). The mixture contained seven enzymes, including proteases, polysaccharidases, lipases, and DNases, and was expected to degrade the major components of biofilm EPS. Some base formulations (e.g., B1K and B1O) did not remove biofilm with or without enzymes. Others (e.g., B2L, B2H, and B2O) also did not remove much biofilm without enzymes (<50%), but the addition of enzymes allowed a strong increase in biofilm removal but only up to a maximum of ∼85%. The best base formulations (B2A, B2B, B2D, and B2I) displayed ∼60% removal without enzymes and 90% removal in combination with enzymes. Other base formulations that exhibited good efficiency of >60% removal without enzymes (e.g., B2C) were not further investigated due to the lesser effect of the enzymes (only 86% removal in combination with enzymes).

The selected base formulations were further optimized by a slight adaption of the detergent composition. In some cases, performance was slightly increased (e.g., B3A compared to B2A), while in other cases, less biofilm was removed (e.g., B3B compared to B2B) (Fig. 1b). B2A, B2B, B3A, B3D2, and B3I2 were identified as the most promising formulations and were further investigated. To study the capacity of the base formulations to support enzyme activities to remove biofilm at reduced enzyme numbers and concentrations, single enzymes (E1 to E8 [one protease, one lipase, one DNase, and five different polysaccharidases]) were individually added at three different concentrations. While all tested base formulations containing E1, E2, or E8 displayed strong biofilm removal ability, only certain formulations supported E3, E4, E5, and E7 activities, and E6 did not remove biofilm in any formulation (see Fig. S1 in the supplemental material). Base formulations B3A and B3D2 allowed significant biofilm removal with six out of the eight enzymes at low enzyme concentrations (0.5% enzyme solution in the cleaner concentrate), whereas the other base formulations supported fewer enzymes. With an increase of the enzyme concentration to 2.5%, biofilm removal was increased for several enzymes in B3A but not in B3D2. Thus, B3A was selected for the following optimization of cleaner composition.

### Optimization of enzyme composition.

**FIG 2:**
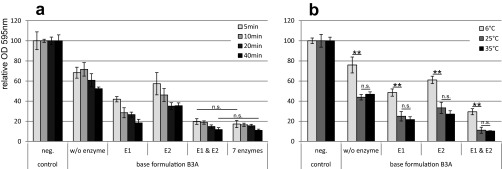
Biofilm removal under different conditions. P. aeruginosa biofilm was treated for different time periods at 25°C (a) or at different temperatures for 40 min (b) with base formulation B3A containing no, one, or two enzymes. The *y* axis represents the biofilm amount quantified by crystal violet staining relative to the negative control. Error bars represent results from 6 individual replicates. Enzyme E1 represents a polysaccharidase, and enzyme E2 is a protease. A *t* test was applied to calculate statistical significance (not significant [n.s.; *P* > 0.05] or highly significant [**, *P* < 0.001]) for comparisons, as indicated by lines in the graph.

Since enzyme activities are strongly dependent on treatment duration and temperature, different conditions were investigated. For base formulation B3A with single enzymes, a clear increase in the biofilm removal efficiency was observed with an increase of the incubation time from 5 to 40 min (Fig. 2a); thus, an incubation time of 40 min was selected. Treatment at both 25°C and 35°C resulted in good biofilm removal, while at 6°C, the cleaner was clearly less efficient (Fig. 2b). This demonstrates that a temperature of 25°C is sufficient for good performance in biofilm removal, which is important for a manual cleaner that is usually used at room temperature.

Investigation of different combinations of enzymes selected from a total of 13 individual enzymes (2 proteases, 9 polysaccharidases, 1 lipase, and 1 DNase) in base formulation B3A revealed that a mixture of 2 enzymes (1 polysaccharidase, E1, and 1 protease, E2 [0.5% {vol/vol} of the concentrate each]) was sufficient to remove 90% of the P. aeruginosa biofilm within 40 min, with 80% removal already after 5 min (Fig. 2a). This performance was similar to that of the seven-enzyme mixture. The addition of further enzymes to the two-enzyme mixture did not increase biofilm removal significantly. Enzyme mixtures missing either E1 or E2 were not able to reach similar levels of biofilm removal. However, based on the performance against artificial blood contaminations, two additional enzymes were included in base formulation B3A, and enzyme concentrations were increased. For example, improvement in cleaning of TOSI slides was observed with increasing concentrations of E2, representing a protease (see Fig. S2 in the supplemental material). The final cleaner containing four enzymes (1 to 2% each) is named deconex Prozyme Active.

### Comparison of different cleaners.

**FIG 3:**
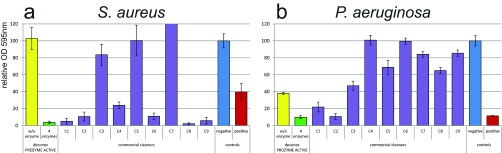
Removal of S. aureus (a) and P. aeruginosa (b) biofilms with different cleaners. Nine commercial cleaners (purple) were compared to deconex Prozyme Active (green) and its corresponding base formulation B3A without enzymes (yellow). The *y* axis represents the biofilm amount quantified by crystal violet staining relative to the WSH-treated negative control (blue). A mixture of 1% SDS, 1% EDTA, 1% NaOH, and 0.1% NaClO was used as a positive control (red). Error bars represent results from 6 individual replicates.

The novel formulation deconex Prozyme Active was compared with 9 comparable commercially available cleaners from different manufacturers (see Table S1 in the supplemental material). Staining of the total biomass with crystal violet revealed that S. aureus was removed easily by most commercial cleaners, including deconex Prozyme Active, that contain a protease (Fig. 3a). This is consistent with data from previous reports (24, 25). The tested nonenzymatic cleaners (C5 and C7) were not able to remove S. aureus biofilms under the static conditions used, and the positive control also only partially (60%) removed the biofilm.

In contrast, the positive control was effective against the P. aeruginosa biofilm, but most cleaners were less efficient in removing this biofilm (Fig. 3b). A mixture of several enzymes in combination with an effective base formulation was required to reach appropriate removal. deconex Prozyme Active removed up to 90% of the P. aeruginosa biofilm, which is slightly better than the positive control and similar to the best commercial cleaner, C2. With 80% biofilm removal, C1 was also efficient, but the rest of the products removed <50% of the biofilm. To differentiate the cleaning and killing activities of the cleaners, the viability of the remaining cells after cleaning was further analyzed by using the BacTiter-Glo assay. Remaining viable cells were at levels similar to remaining biomass after treatment with most cleaners (<0.8-log difference between viable cells and biomass [indicated by dashed bars in Fig. S3 in the supplemental material]). Thus, these cleaners did not possess substantial biocidal activity. In contrast, it was found that after treatment with C3 and C7, substantially fewer viable cells (in percentage) were found than the remaining biomass (>1.8-log difference). These cleaners displayed disinfecting properties and rather killed bacteria instead of removing the biofilm.

The efficiency of deconex Prozyme Active in removing artificial blood contamination was also compared to that of commercial products (see Fig. S4 in the supplemental material). Only C2 performed slightly better than and C1 and C8 performed similarly to the new formulation, while all the other commercial cleaners required longer incubation times to reduce and remove the soil. Two nonenzymatic cleaners, C5 and C7, displayed the lowest activity against this artificial blood soil.

### Performance against biofilm in endoscope channels.

So far, there is not a standard procedure for testing biofilm removal with manual cleaners. In the technical specification Annex F of part 5 in EN ISO 15883 (version 2005) (https://www.iso.org/obp/ui/#iso:std:iso:ts:15883:-5:ed-1:v1:en), a method for biofilm formation and evaluation of biofilm removal from endoscope channels is described. This method is used for testing cleaners and disinfectants in automated processes at elevated temperatures (usually 35°C to 55°C). Acceptance criteria for this method for biofilm cleaning efficacy are set at 90% removal of proteins and polysaccharides. This procedure was used for testing deconex Prozyme Active and other manual cleaners for their biofilm removal capabilities during 15 min of treatment under a continuous flow of 200 ml/min.

With deconex Prozyme Active, the CFU of the P. aeruginosa biofilm were reduced by >2 logs (99%) compared to WSH-treated control tubes (see Fig. S5 in the supplemental material). Clearly, more biofilm was removed if the cleaner contained enzymes than when the cleaner contained an enzyme-free base formulation, demonstrating the beneficial effect of the enzymes. Commercial cleaners C4 (0.28-log reduction) and C6 (0-log reduction) did not sufficiently reduce CFU, while C1 (1.62-log reduction) and C2 (1.51-log reduction) were slightly less effective than the novel formulation (2.11-log reduction). After treatment with C7, almost no viable bacteria were recovered (5.57-log reduction). The results regarding the efficiency of removal of EPS compounds were similar to those for the remaining CFU, except for C7, where neither protein nor polysaccharide levels were significantly reduced (see Fig. S6 in the supplemental material). This suggests that C7 killed the bacteria rather than removing the biofilm, which is consistent with observations from the 96-well-plate biofilm removal assay. These results are summarized in Table 1.

**TABLE 1:** Reduction of biofilm biomass, numbers of viable bacteria, polysaccharide levels, and protein levels after treatment with cleaners compared to the negative-control (WSH) treatment^*d*^

Cleaner	% reduction of biomass as determined by OD_600_	% reduction of bacterial CFU	Log reduction of bacterial CFU^*a*^	% reduction in polysaccharide levels^*b*^	% reduction in protein levels^*c*^
Base formulation B3A	82.3	89.21	0.97	79.2	82.4
deconex Prozyme Active	94.6	99.23	2.11	93.1	97.9
C1	93.0	97.61	1.62	86.2	89.4
C2	91.4	96.89	1.51	84.6	95.1
C6	−10.2	−1.32	−0.01	−31.5	−9.2
C4	19.4	47.81	0.28	19.2	19.0
C7	−34.4	99.99973	5.57	13.1	5.6

a Log_10_ reduction compared to the negative control.

b As determined by the phenol-sulfuric acid method described previously by Dubois et al. (23).

c As determined by the protein quantification assay described previously by Lowry et al. (22).

d Negative values indicate a lower level of removal than with WSH.

Another important criterion for the standard assay is that cleaning should allow complete killing of all bacteria by subsequent disinfection. Therefore, the biofilm remaining on the tube was subsequently treated with a high-level disinfectant (deconex HLD PA/PA20). No viable bacteria were recovered from the disinfected deconex Prozyme Active-treated tubes, while ∼1,600 CFU per cm^2^ were found on tubes treated with WSH (negative control) prior to disinfection. This demonstrates the importance of an efficient cleaning step to enable the success of the consequent disinfection.

**FIG 4:**
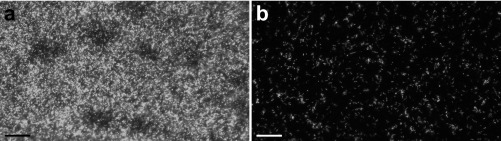
Bacteria remaining on endoscope tubes after treatment. Syto9 staining of P. aeruginosa biofilm on tubes treated with WSH (a) or deconex Prozyme Active (b) was visualized with a 20× water immersion objective. Bacterial cells appear as white spots. Bars, 25 μm.

Microscopy analysis was performed to confirm biofilm cleaning. It was observed that large parts of the biofilm were removed by treatment with deconex Prozyme Active but not after WSH treatment. While the control displayed a dense biofilm with multiple layers (99.6% surface coverage), the deconex Prozyme Active-treated sample exhibited much lower surface coverage (11.1%), and almost no aggregates were observed (Fig. 4). deconex Prozyme Active was also found to be superior to the other cleaners regarding biofilm removal from endoscope tubes (see Fig. S7 in the supplemental material). Only C1 (45.8% coverage) and C2 (27.4% coverage) also displayed some bacterium-free areas, but in addition to higher surface coverage, more aggregates were observed. For C7, bacteria appeared blurry, even though the cells were perfectly in focus. This was likely due to killing of the bacteria by destroying their membrane integrity, as a similar effect was observed when the WSH-treated tube was disinfected with deconex HLD PA/PA20 (see Fig. S8 in the supplemental material).

### Stability of the formula regarding cleaner performance.

To investigate the stability of the formulation, the cleaner concentrates were stored at room temperature (25°C) and, for accelerated aging, at increased temperatures. After storage for 24 weeks at 25°C, the new formulation still removed >90% of the S. aureus biofilm and >85% of the P. aeruginosa biofilm (see Fig. S9 in the supplemental material). Storage of the cleaner concentrates at 40°C did not affect the performance significantly. Even after incubation at 50°C for 24 weeks, ∼75% of the biofilm was removed, being clearly more effective than the enzyme-free version, which removed <50% of the biofilm. Additionally, artificial blood contaminations were removed effectively by deconex Prozyme Active stored at 25°C and 40°C for 24 weeks, while at 50°C, its activity was impaired slightly after 12 weeks and slightly more after 24 weeks (data not shown). This confirms that the product keeps its activity during storage at room temperature and even survives short periods at higher temperatures, e.g., during transport.

### Conclusions.

The 96-well-plate biofilm removal assay and the endoscope ISO test led to matching results regarding the efficiency of the novel and commercially available cleaners studied. For total biofilm biomass assessment, the results of optical density, protein, and polysaccharide quantification with the ISO test correlated with the results of crystal violet staining in the 96-well-plate assay, while for viable bacteria, the CFU corresponded to those determined by the BacTiter-Glo assay. This confirms that the 96-well-plate assay represents an appropriate model to screen for cleaners that remove biofilms and to investigate which formulation rather acts as a disinfectant.

The addition of enzymes to the base formulation had a clear beneficial effect on the efficiency of biofilm removal. The S. aureus biofilm was removed efficiently if an active protease was present, whereas for P. aeruginosa, single enzymes added to the formulation were not sufficient. An optimized enzyme mixture including protease, polysaccharidases, and other enzymes in a selected base formulation was required to achieve efficient removal of P. aeruginosa. Therefore, many commercial products displayed good performance against S. aureus and blood contamination but had problems with the removal of P. aeruginosa biofilms. Nonenzymatic cleaners were not effective in either blood cleaning or biofilm removal but rather worked as a disinfectant, killing the bacteria. However, a cleaner should mainly remove bacteria, as the standard endoscope reprocessing procedure is followed by disinfection. Among the tested high-end enzymatic endoscope detergents, the novel cleaner deconex Prozyme Active demonstrated the best efficiency in biofilm removal. Additionally, it was among the best products in removing blood contamination.

## Supplementary Material

Supplemental material
