# Supplementary material for: Enzymes Enhance Biofilm Removal Efficiency of Cleaners
Source: Antimicrob Agents Chemother. 2016 May 23;60(6):3647–52. doi: 10.1128/AAC.00400-16 (PMC4879406; doi:10.1128/AAC.00400-16)
Supplement: Supplemental material [file supp_60_6_3647__index.html]

Enzymes Enhance Biofilm Removal Efficiency of Cleaners — Supplemental material 

# Enzymes Enhance Biofilm Removal Efficiency of Cleaners

## Supplemental material

- Supplemental file 1 -

  Additional experimental details, Table S1, and Figures S1 to S9.

  PDF, 2.0M
