# Supplementary material for: Enzymes Enhance Biofilm Removal Efficiency of Cleaners
Source: Antimicrob Agents Chemother. 2016 May 23;60(6):3647–52. doi: 10.1128/AAC.00400-16 (PMC4879406; doi:10.1128/AAC.00400-16)
Supplement: Supplemental material [file AAC.00400-16_zac006165242so1.pdf]

Supplementary Data

Enzymes enhance biofilm removal efficiency of cleaners

Philipp Stiefel[1], Stefan Mauerhofer[2], Jana Schneider[1], Katharina Maniura-Weber[1], Urs Rosenberg[2], Qun Ren[1]*

[1] Laboratory for Biointerfaces, Empa, Swiss Federal Laboratories for Materials Science and Technology, Lerchenfeldstrasse 5, CH-9014 St. Gallen, Switzerland

[2] Borer Chemie AG, Gewerbestrasse 13, 4528 Zuchwil, Switzerland

* Corresponding author
E-mail: qun.ren@empa.ch

Material & Methods

Cleaning performance using EN ISO 15883

Biofilm was formed in a system according to ISO/TS 15883-5:2005(E) Annex F with slight adaptions. Polytetrafluoroethylene (Teflon) tube (KARL STORZ, Germany) with a diameter of 4 mm was used, and flow rates of 1 ml/min inlet and 40 ml/min circling were applied. The whole system was placed in an oven at 30°C instead of using a water bath described in EN ISO 15883. A 2.5 m long Teflon tube was used per experiment. *P. aeruginosa* DSM No. 1117 was used instead of the *P. aeruginosa* strains described in the norm. Biofilm was grown for 72 hours.

Tubes were cut into 30 mm parts and rinsed with 0.9% NaCl solution for 1 minute with peristaltic pump (~20 ml/min). Treatment with cleaner or WSH (negative control) was done with ~200 ml/min flow for 15 minutes at 25°C. Disinfection (only done if indicated) after cleaning was done with deconex® HLD PA / PA20 (Borer Chemie AG) flowing through the 30 mm tube parts for 15 min using a peristaltic pump (~5 ml/min). The tube parts were rinsed with 0.9% NaCl solution for 1 minute with peristaltic pump (~20 ml/min). The tubes parts were cleaned outside with a 70% EtOH containing paper towel and cut longitudinally into half and into 5 mm pieces. The small pieces were added to a Falcon tube containing 20 ml 0.9% NaCl solution and vortexed for 5 minutes to detach the bacterial cells.

The following quantifications were done:

1) Total biomass cells were measured by optical density of the suspension at 600 nm

2) Viable cells were quantified by plating dilution series
A 1:5 dilution series (30 µl in 120 µl 0.9% NaCl solution) was prepared in 96-well plates down to a dilution of 5^{-11}. 5 µl of each sample and dilution were pipetted onto TSA square plates. Colonies were counted after 1 day incubation at 25°C.

3) Proteins were quantified by Lowry assay (1)
Complex-forming reagent was prepared immediately before use by mixing the following three stock solutions A, B, and C in the proportion 100:1:1 (v:v:v), respectively: Solution A: 2% (w/v) Na2CO3 in distilled water; Solution B: 1% (w/v) CuSO4·5H2O in distilled water; Solution C: 2% (w/v) sodium potassium tartrate in distilled water. 1 ml sample was mixed with 1 ml of complex-forming reagent and incubated for 10 min. 0.5 ml Folin reagent was added and mixed by vortexing. The mixture was incubated for 30 min before measuring the absorbance at 650 nm.

4) Polysaccharides were quantified by the phenol sulfuric acid method (2)
1 ml of 5% phenol solution was added to a 2 ml sample, afterward immediately 5 ml of 95% sulfuric acid was added. The mixture was incubated for 10 min, carefully shaken and incubated for another 20 min. Absorbance was measure at 490 nm.

Aging of the cleaner concentrates

One liter cleaner concentrate samples of the same batch were placed in ovens at 25°C, 40°C and 50°C, respectively. Higher temperatures should on the one hand simulate accelerated aging conditions and on the other hand reveal if the cleaner endures heating at unfavorable transport and storage conditions. 10 ml samples were taken after different incubation times to determine their performance of biofilm removal in the 96-well plate system and efficiency of cleaning artificial blood contaminations on TOSI® slides as described above.

Microscopy

Biofilm containing endoscope tubes prepared and treated as described above were cut longitudinally into half and into 5 mm pieces. The pieces were individually added to a 12-well plate containing 1 ml 2.5 µM SYTO9 (life technologies) in 0.9% NaCl solution per well. After 30 min of incubation the tube pieces were placed into a Petri dish filled with water. Microscopy pictures were immediately taken using a 20x water objective and GFP filters with the Leica DM6000 B microscope, Leica DFC450 C camera and the Leica LAS AF software. Surface coverage was determined by CellProfiler software identifying objects above an intensity threshold of 0.15.

Results

Selection of conditions for the 96-well plate system

Biofilm formation

Biofilm formation in polystyrene 96-well plates for 24 hours at 33°C and 40 rpm was found to suit best our purpose. Sufficient biofilm of both species was formed for testing the efficacy of cleaner formulations. Additionally, physiological sodium chloride solution (0.9% NaCl solution) and water of standardized hardness (WSH) did not remove the biofilm, whereas the positive control (1% SDS, 1% EDTA, 1% NaOH, 0.1% NaClO) removed a substantial amount of the biofilm. Different temperatures for biofilm formation were investigated. For example, after 24 h more *P. aeruginosa* biofilm was formed at 37°C than at 33°C, however this former biofilm was found to be more easily removed by cleaners. Also biofilm formed for 48 h was investigated which displayed similar biomass quantity and cleaning resistance compared to 24 h incubation. Therefore, longer incubation time was not necessary. Different media were compared and 30% TSB supplemented with additional glucose was selected, because it supported strong biofilm formation of both strains.

Cleaner treatment

It was found that the cleaner removed more biofilm if it was diluted in WSH than in deionized water, especially in combination with enzymes. Static conditions at 25°C for different incubation times of 5, 10, 20 and 40 min were tested. For deconex® PROZYME ACTIVE 5 min were sufficient to display almost maximal performance. However, longer incubation times were selected as the results were more reproducible and most other cleaners were less effective with short incubation times. Different temperatures for the cleaner treatment were also investigated. The performance was clearly reduced at 6°C, while there was no significant difference between 25°C and 35°C. Finally, 25°C and 40 min treatment were selected to simulate the manual cleaning conditions of the endoscope in the cleaner bath at room temperature.

Biofilm quantification

Staining with Crystal Violet was used to investigate the total amount of biomass, while the BacTiter-Glo assay is a sensitive and precise method to quantify the amount of viable cells (3).

References

1. **Lowry OH, Rosebrough NJ, Farr AL, Randall RJ.** 1951. Protein measurement with the Folin phenol reagent. J Biol Chem **193:**265-275.
2. **Dubois M, Gilles K, Hamilton JK, Rebers PA, Smith F.** 1951. A colorimetric method for the determination of sugars. Nature **168:**167.
3. **Stiefel P, Rosenberg U, Schneider J, Mauerhofer S, Maniura-Weber K, Ren Q.** 2016. Is biofilm removal properly assessed? Comparison of different quantification methods in a 96-well plate system. Appl Microbiol Biotechnol **AMAB-D-15-02128R3 [accepted]**.

Table S1: Commercial high-end cleaners with similar specifications.

Cleaner	Enzymes [a]	Claim on biofilm removal [b]	Specifications [c]
**C1**	protease, lipase, amylase	yes	manual cleaning of instruments (especially endoscopes) at low temperature
**C2**	protease, amylase, cellulase	yes	manual cleaning of instruments (especially endoscopes) at low temperature
**C3**	protease, several others - not specified	none	manual cleaning of instruments (especially endoscopes), containing disinfecting properties
**C4**	protease, lipase, amylase, cellulase, mannanase	yes	manual and automated cleaning of instruments at low temperature, low foaming
**C5**	none (enzyme-free cleaner)	yes	manual cleaning of instruments (including endoscopes) at low temperature, low foaming
**C6**	protease	yes	manual cleaning of instruments (especially endoscopes) at low temperature
**C7**	none (enzyme-free cleaner)	yes	manual and automated cleaning of instruments (especially endoscopes)
**C8**	proteases	none	cleaning of instruments (especially endoscopes), low foaming
**C9**	protease, amylase, cellulase, 1 more not specified	yes	manual cleaning of instruments (including endoscopes)

[a] all enzymes specified in manufactures' brochures or safety datasheets

[b] statements found in official brochures or on internet sites of the manufacturer

[c] information provided by the manufacturer

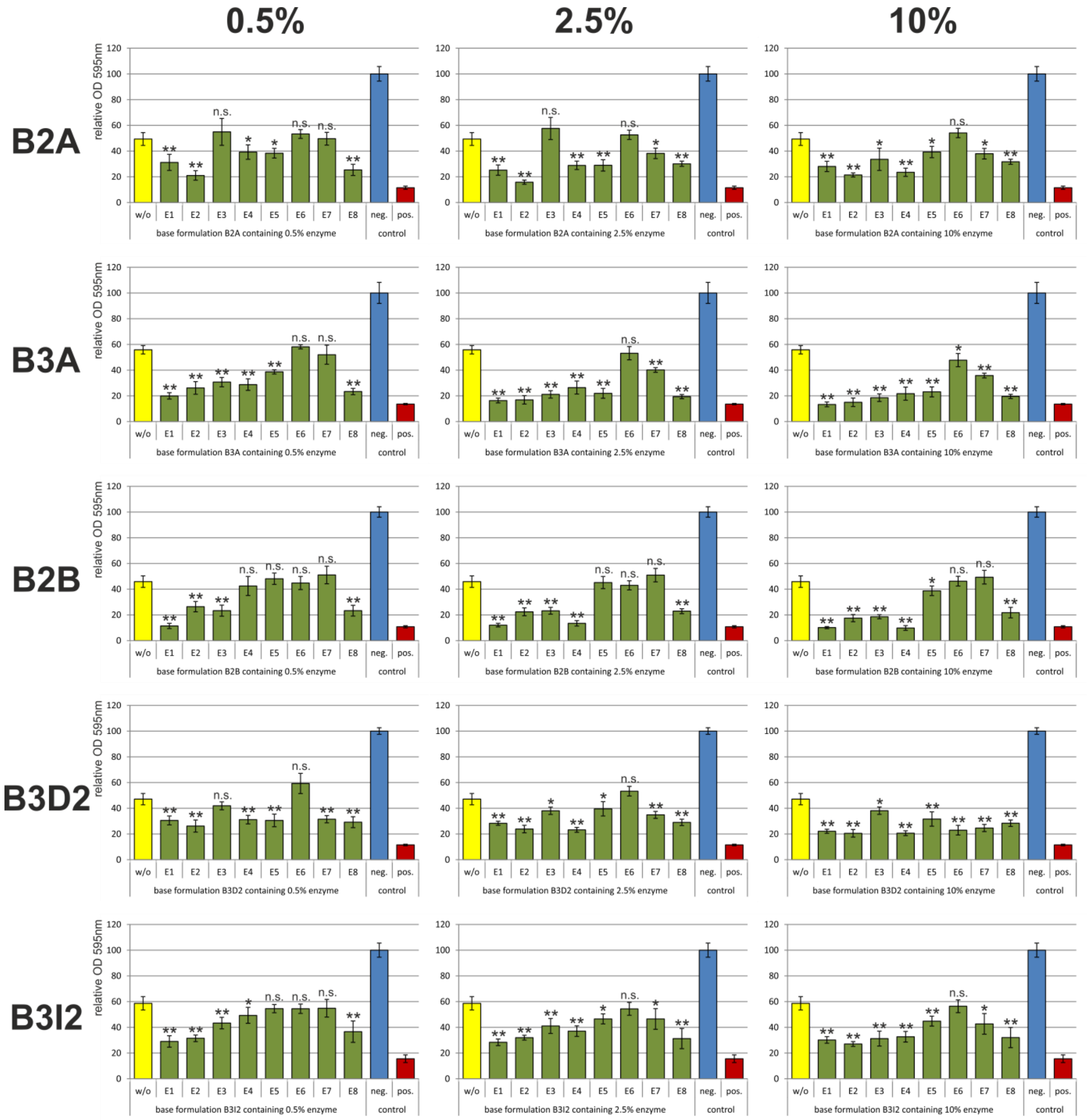

Figure S1: *P. aeruginosa* biofilm removal with selected novel base formulations containing single enzymes. Different enzyme concentrations of 0.5%, 2.5% and 10% were added to the cleaner concentrate. Base formulation without enzyme (yellow) was used as comparison. Y-axis represents the biofilm amount quantified by Crystal Violet staining relative to the negative control. Error bars represent 6 individual replicas. A *t*-test was applied to each enzyme containing cleaner compared to its base formulation to calculate if the differences are statistically not significant (n.s., $p>0.05$) significant (*, $p<0.05$) or highly significant (**, $p<0.001$).

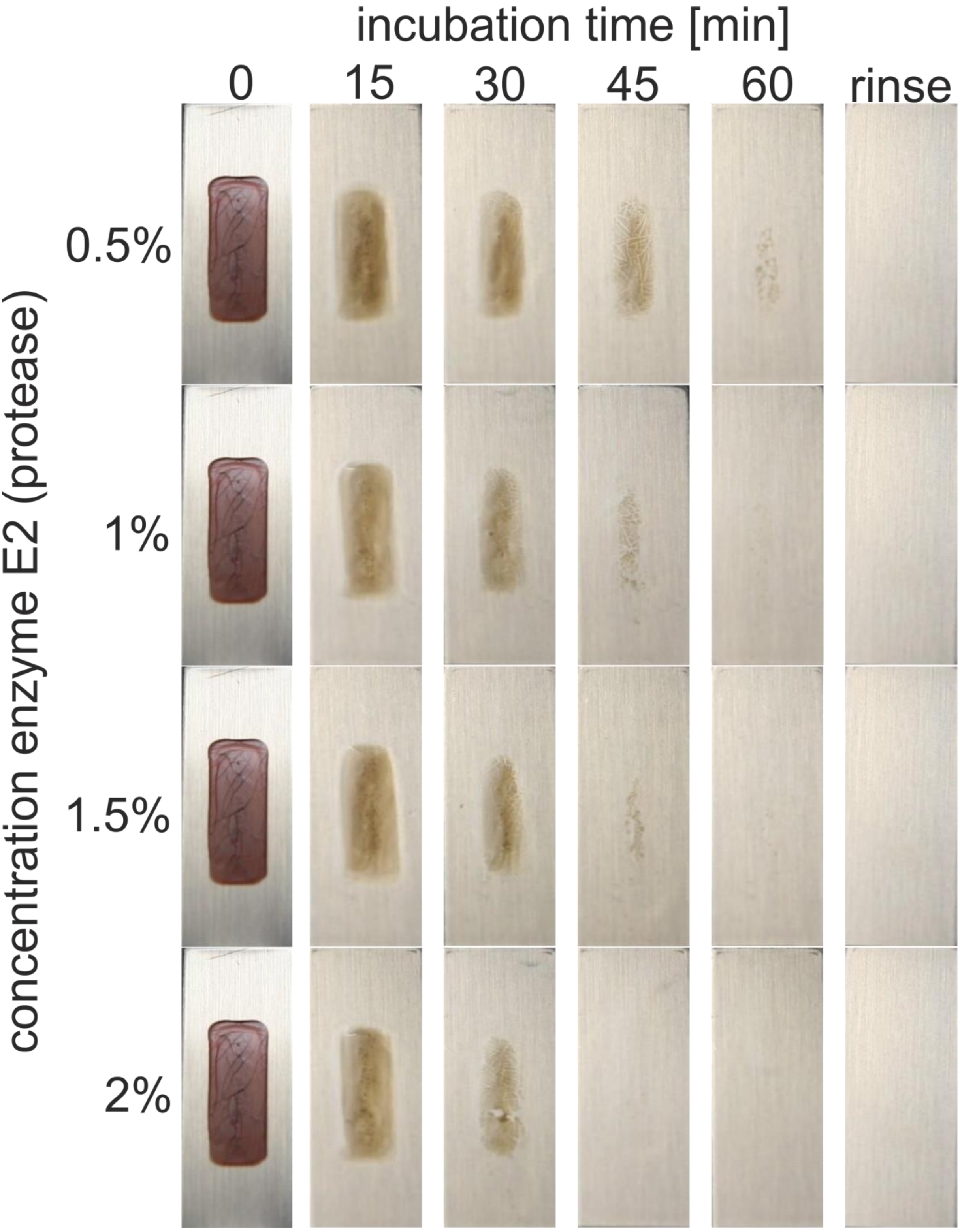

Figure S2: Dependence of protease concentration on artificial blood cleaning. TOSI® slides were treated with deconex® PROZYME ACTIVE with varying concentrations of protease, while the other three enzymes were at constant level. Percent specifications indicate the protease concentration relative to the cleaner concentrate. Pictures of remaining artificial blood soil after 0, 15, 30, 45, 60 minutes and after additional rinsing (left to right) are displayed.

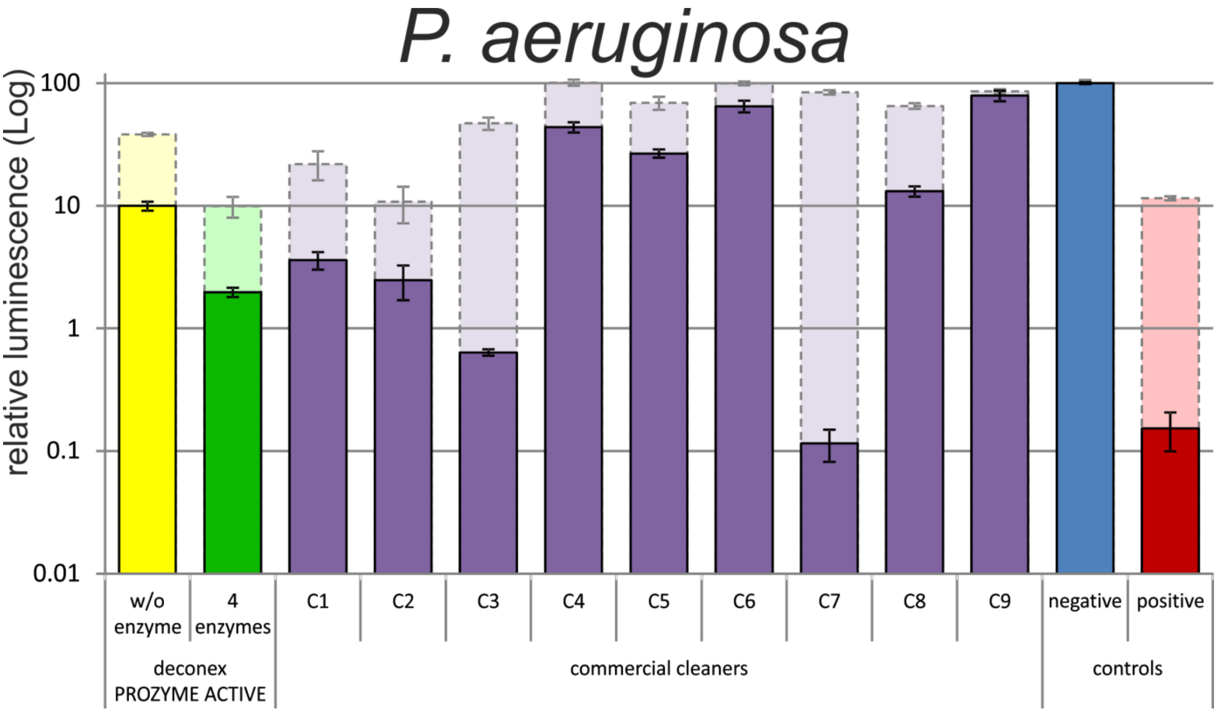

Figure S3: Viability of remaining biofilm after treatment with different cleaners. 9 commercial cleaners (purple) are compared to deconex® PROZYME ACTIVE (green) and its corresponding base formulation B3A without enzymes (yellow). Viable bacteria of *P. aeruginosa* were quantified by BacTiter-Glo assay. Y-axis represents the viability relative to the negative control presented in Log scale. Error bars represent the values generated from 6 individual wells. For better comparison of killing and removal activities the data of biomass quantification from Figure 3 are also indicated in the background with dashed lines.

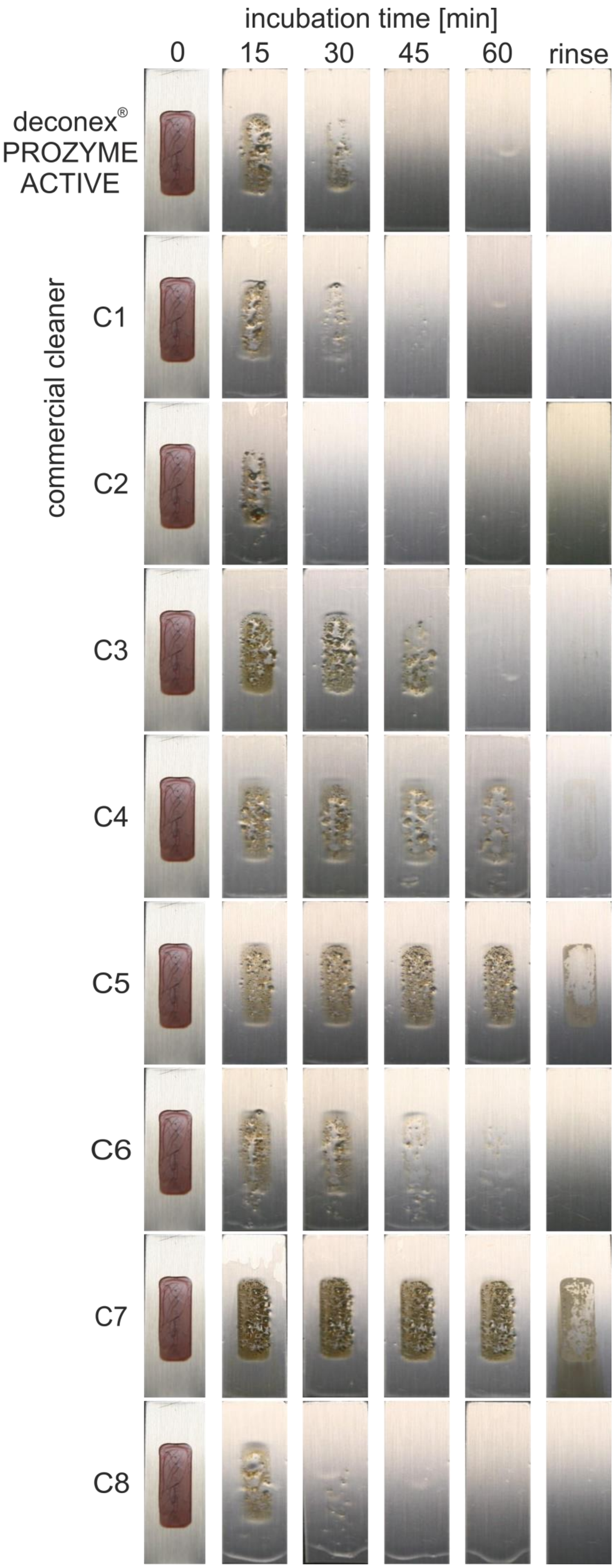

Figure S4: Artificial blood cleaning with different cleaners. TOSI® slides were treated with the different cleaners diluted to 1% in WSH. 8 commercial cleaners were compared to deconex® PROZYME ACTIVE. Pictures of remaining artificial blood soiling after 0, 15, 30, 45, 60 minutes and after additional rinsing (left to right) are displayed.

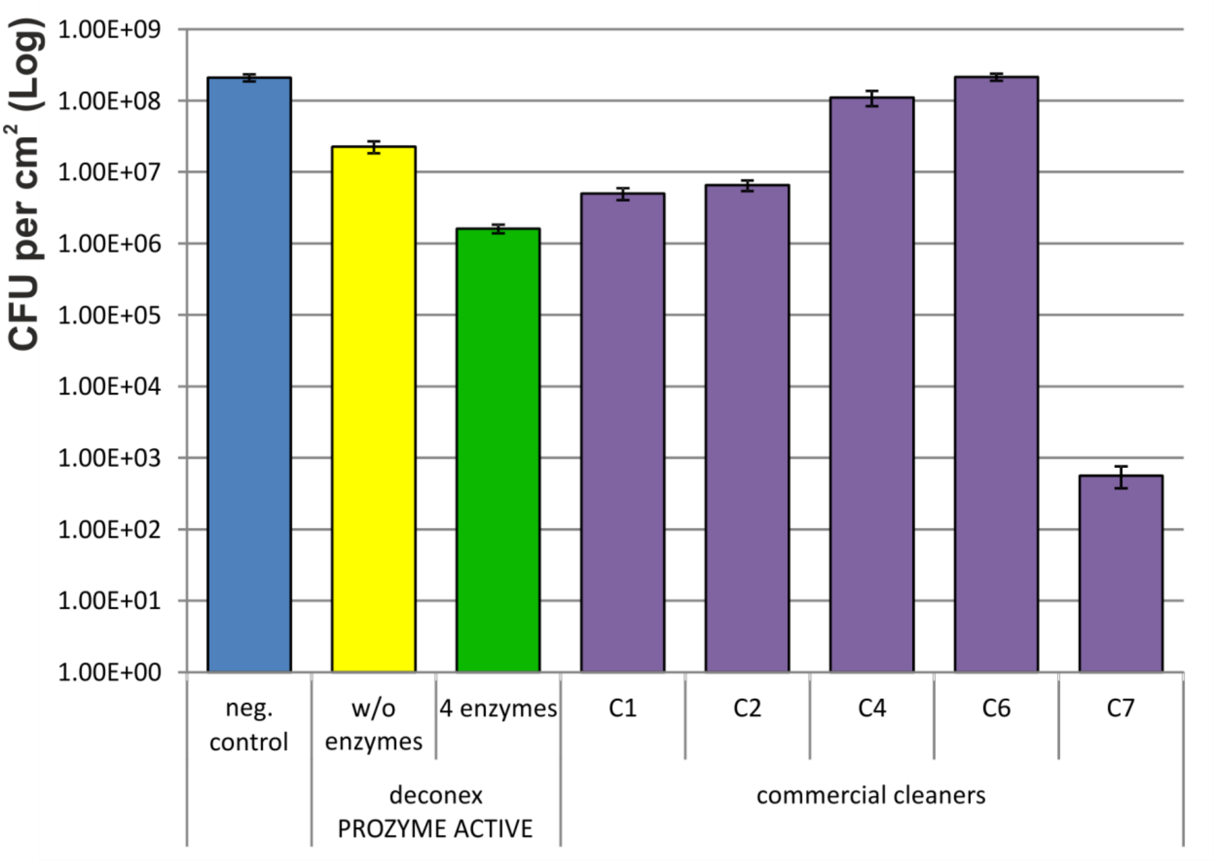

Figure S5: Colony forming units after treatment with different cleaners. Bacterial cells were released from endoscope tubes after treatment with different cleaners and dilution series were made and cells were grown on agar plates. CFU were counted to calculate viable bacteria per square centimeter of the tube. 5 commercial cleaners (purple) were compared to deconex® PROZYME ACTIVE (green) and its corresponding base formulation B3A without enzymes (yellow). Standard deviations represent 3 individual dilution series, each plated 3 times.

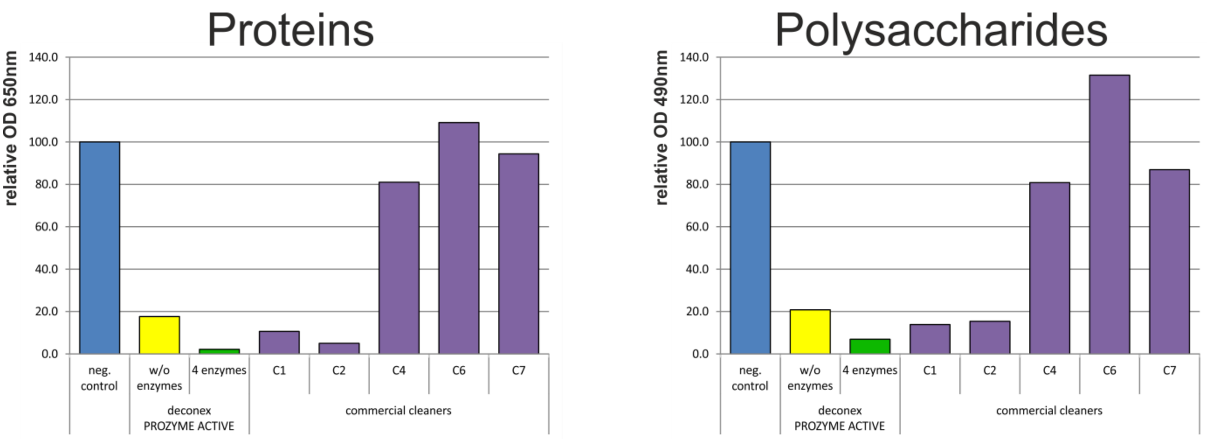

Figure S6: EPS compounds after treatment with different cleaners. Biofilm was released from endoscope tubes after treatment with different cleaners. Proteins **(a)** were quantified by the Lowry method and polysaccharides **(b)** by the Dubois method. Y-axis represents the values relative to the WSH treated negative control. 5 commercial cleaners (purple) were compared to deconex® PROZYME ACTIVE (green) and its corresponding base formulation B3A without enzymes (yellow).

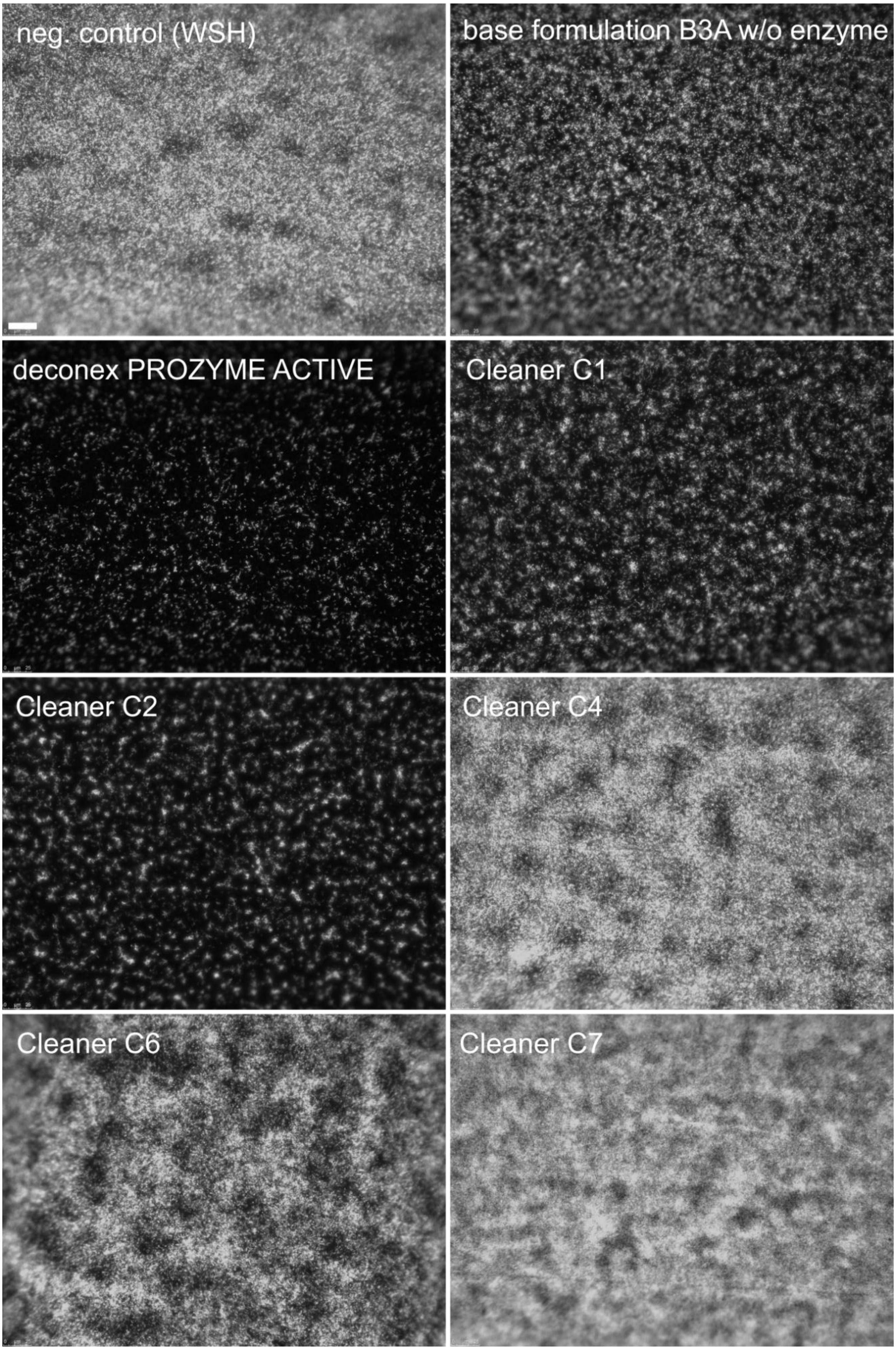

Figure S7: Remaining biofilm on the tubes. Syto9 stained bacteria on tubes treated with different cleaners and observed with 20x water objective. Bacteria appeared as white spots. The scale bar is 25µm.

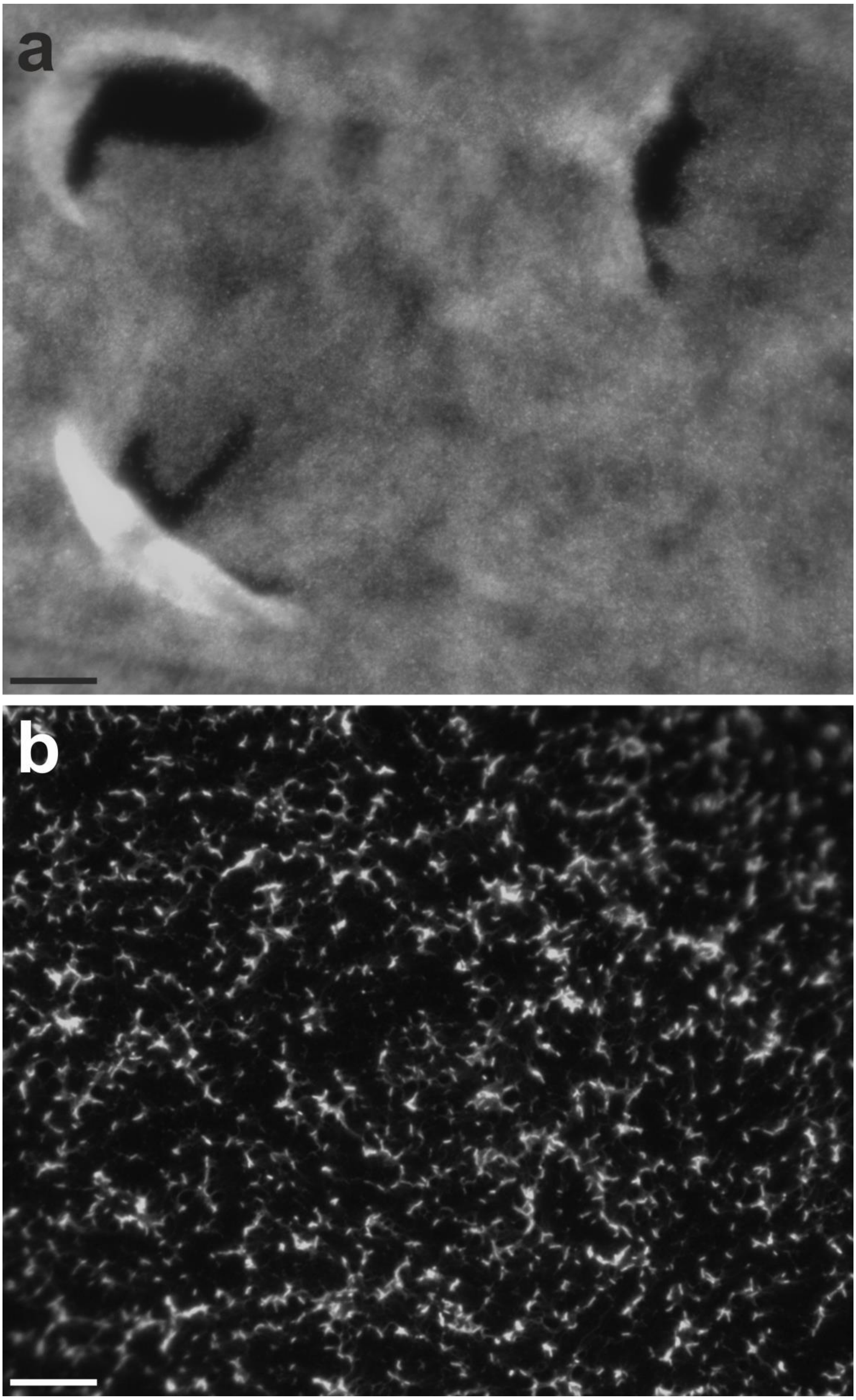

Figure S8: Remaining biofilm on the tubes after disinfection. Syto9 stained bacteria after disinfection of tubes previously treated with WSH **(a)** or deconex® PROZYME ACTIVE **(b)**. Tubes were observed with 20x water objective. The scale bar is 25µm.

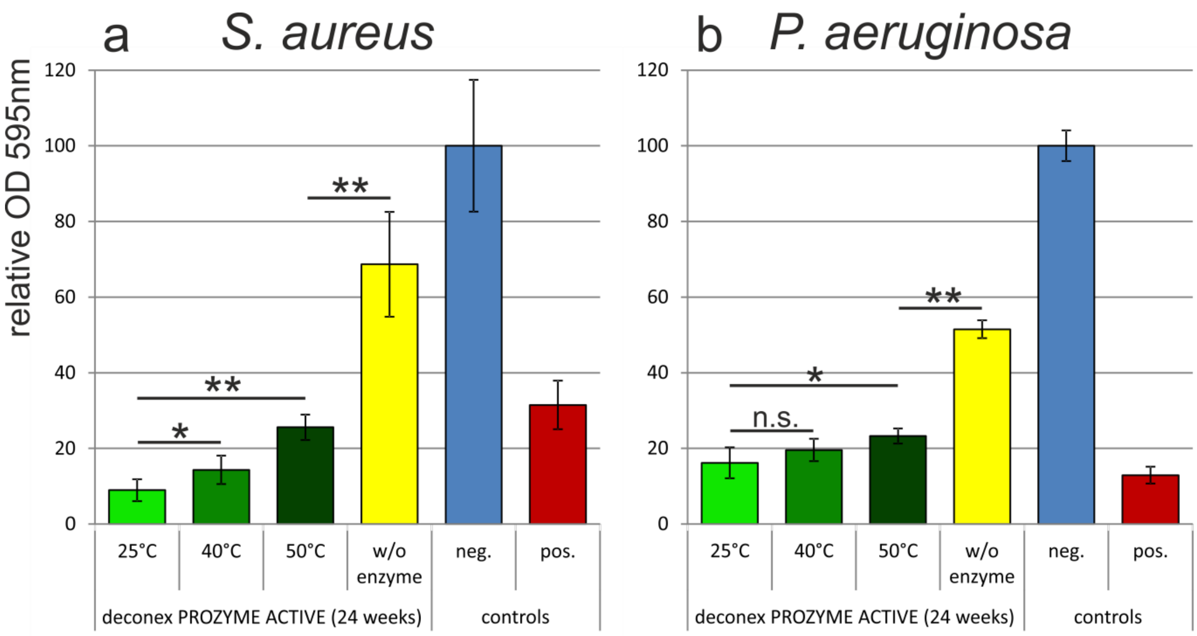

Figure S9: Biofilm removal upon accelerated aging of the cleaners. Remaining *S. aureus* **(a)** and *P. aeruginosa* **(b)** biofilm after treatment with cleaners incubated at different temperature for 24 weeks. deconex® PROZYME ACTIVE concentrate was either stored at 25°C (light green), 40°C (green) or 50°C (dark green). Base formulation B3A without enzymes (yellow) and positive control (red) are also displayed. Y-axis represents the biofilm amount quantified by Crystal Violet staining relative to the negative control (blue). Error bars represent the values obtained from 6 individual wells. A *t*-test was applied to calculate if the differences are statistically not significant (n.s., $p>0.05$) significant (*, $p<0.05$) or highly significant (**, $p<0.001$).
